# Extracellular vesicles containing ACE2 efficiently prevent infection by SARS‐CoV‐2 Spike protein‐containing virus

**DOI:** 10.1002/jev2.12050

**Published:** 2020-12-28

**Authors:** Federico Cocozza, Nathalie Névo, Ester Piovesana, Xavier Lahaye, Julian Buchrieser, Olivier Schwartz, Nicolas Manel, Mercedes Tkach, Clotilde Théry, Lorena Martin‐Jaular

**Affiliations:** ^1^ INSERM U932 Institut Curie Centre de Recherche PSL Research University Paris France; ^2^ Université de Paris Paris France; ^3^ Virus and Immunity Unit Institut Pasteur and CNRS UMR 3569 Paris France

**Keywords:** ACE2, EV therapy, SARS‐CoV‐2, TMPRSS2

## Abstract

SARS‐CoV‐2 entry is mediated by binding of the spike protein (S) to the surface receptor ACE2 and subsequent priming by host TMPRSS2 allowing membrane fusion. Here, we produced extracellular vesicles (EVs) exposing ACE2 and demonstrate that ACE2‐EVs are efficient decoys for SARS‐CoV‐2 S protein‐containing lentivirus. Reduction of infectivity positively correlates with the level of ACE2, is much more efficient than with soluble ACE2 and further enhanced by the inclusion of TMPRSS2.

## 1 INTRODUCTION

SARS‐CoV‐2 is the infectious causative agent of the COVID‐19 pandemic (Zhou et al., 2020). Viral entry into host cells is mediated by the interaction of the spike (S) protein on the surface of SARS‐CoV‐2 with the surface receptor angiotensin‐converting enzyme 2 (ACE2) (Walls et al., 2020). After binding to ACE2, the S protein is cleaved by Transmembrane protease serine 2 (TMPRSS2) and becomes fusogenic thus allowing viral entry (Hoffmann et al., 2020).

ACE2 is expressed at the surface of pneumocytes and intestinal epithelial cells which are potential target cells for infection (Ziegler et al., 2020). A soluble form of the ACE2 ectodomain can be released after cleavage by ADAM10 or ADAM17 in different physiological conditions (Jia et al., 2009). In addition, TMPRSS2 can compete with the metalloproteases for ACE2 cleavage (Heurich et al., 2014). Soluble recombinant ACE2 neutralizes SARS‐CoV‐2 by binding the S protein and has been proven to reduce entry of SARS‐CoV‐2 into Vero‐E6 cells and engineered human organoids (Monteil et al., 2020). ACE2, however, is synthesized as a transmembrane protein, like TMPRSS2. We postulate that ACE2 could be present on the surface of extracellular vesicles (EVs), which could result in better efficacy as a decoy to capture SARS‐CoV‐2. Furthermore, concomitant presence of an active serine‐protease TMPRSS2 on the same EVs could further interfere with viral infectivity, by forcing viral fusion on the EV membrane, rather than on target cells.

EVs are lipid bilayer‐enclosed structures containing transmembrane proteins, membrane‐associated proteins, cytosolic proteins and nucleic acids that are released into the environment by different cell types (Mathieu et al., 2019). Since EVs have the same membrane orientation as cells, they expose at their surface the extracellular domains of transmembrane proteins that can bind to nearby or long‐distance targets. By specifically binding to different proteins and protein‐containing structures, EVs can act as a decoy for virus (De Carvalho et al., 2014) and bacterial toxins (Keller et al., 2020), thus suggesting a potential role as therapeutic agents.

## 2 RESULTS

**FIGURE 1:**
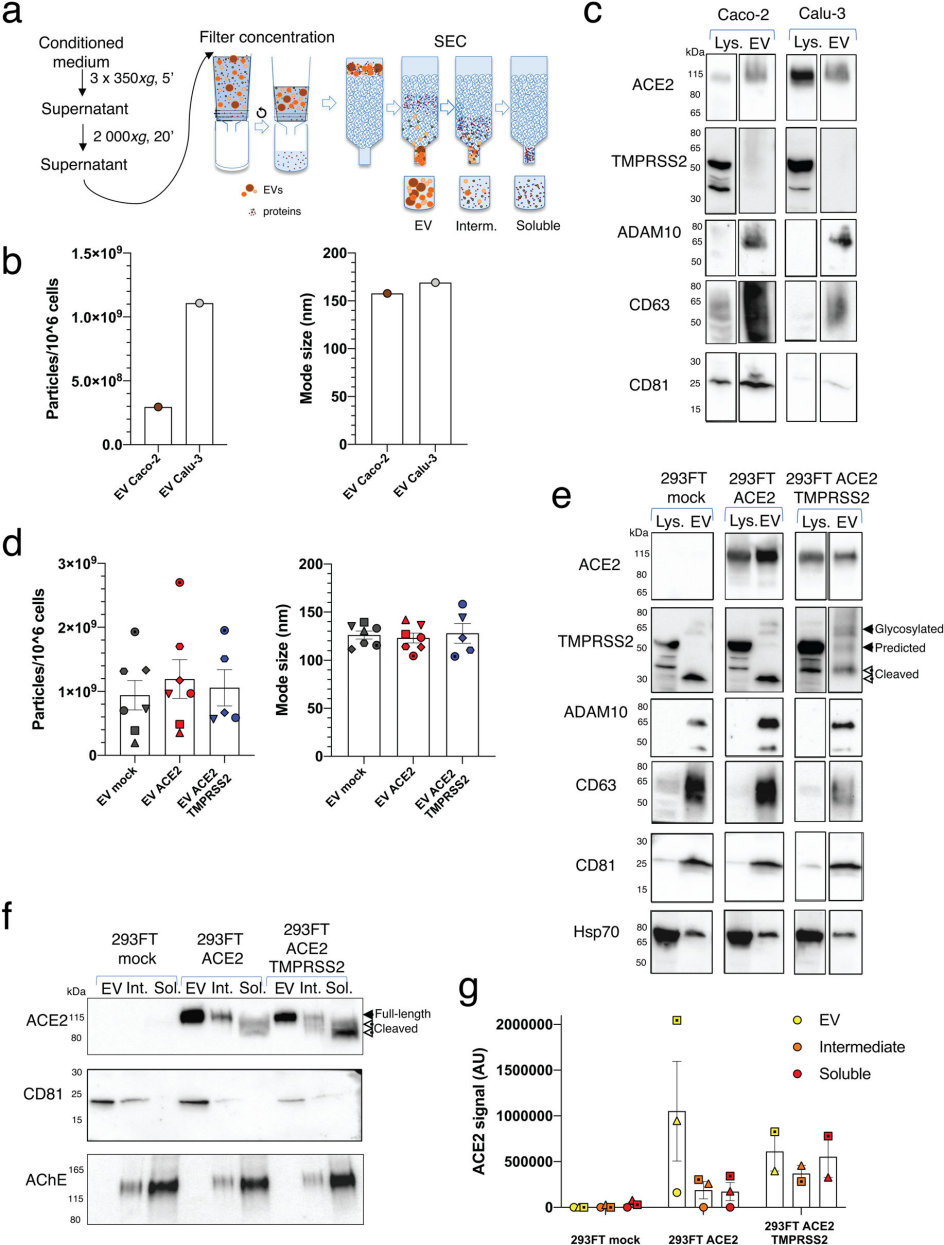
Isolation and characterization of EVs containing ACE2 and TMPRSS2. (a) Scheme of EV isolation and separation from soluble components by SEC. (b) NTA quantification and mode size of the particles in EV‐containing SEC fractions obtained from Caco‐2 and Calu‐3 cells. (c) WB analysis of ACE2, TMPRSS2 and different EV markers in lysates from 4 × 10^5^  cells and 1 × 10^10^  2020 (Caco‐2) or 0.5 × 10^10^ 2020 (Calu‐3) particles obtained from EV SEC fractions. One experiment. (d) NTA quantification and mode size of the particles in EV‐containing fractions obtained from 293FT‐mock, 293FT‐ACE2 and 293FT‐ACE2‐TMPRSS2 cell lines. Different symbols correspond to independent experiments. Error bars indicate SEM. (e) WB analysis of ACE2, TMPRSS2 and different EV markers in lysates from 4 × 10^5^ cells and 1 × 10^10^ 2020 particles from EV SEC fractions obtained from the three 293FT cell lines. (f‐g) WB analysis of ACE2, CD81 and AChE on EV, intermediate and soluble fractions from the three 293FT cell lines. 1 × 10^9^  particles from the EV fraction or material recovered from the same corresponding volume of CCM, for the intermediate or soluble fractions, were loaded on the gel. (f) Representative WB. (g) Quantification of ACE2 signal (pooled full‐length and cleaved forms) in 2–3 independent WB

In order to explore the hypothesis that EVs can be used as SARS‐CoV‐2 decoy agents, we first assessed whether ACE2 can be present in EVs from cell lines derived from tissues expressing ACE2. As cell lines endogenously expressing ACE2, we used the human lung epithelial cell line Calu‐3 and the epithelial colorectal cell line Caco‐2 which are known targets for SARS‐CoV‐2 infection (Hoffmann et al., 2020). In a pilot experiment, Calu‐3 and Caco‐2 were cultured in medium without fetal bovine serum (FBS) for 24 h and EVs were isolated from the cell conditioned medium (CCM) by size‐exclusion chromatography (SEC). This technique allows the separation of EVs from soluble proteins (Figure 1a). We analysed side‐by‐side fractions enriched in EVs (pooled fractions 7–11), in soluble factors (pooled fractions 17–21), and the fractions in‐between (pooled fraction 12–16) (Figure 1a). Particle quantification by nanoparticle tracking analysis (NTA) confirmed that the majority of particles released by Calu‐3 and Caco‐2 cells were isolated in EV‐containing fractions (Supplementary Figure A). In this experiment, Caco‐2 cells released less EVs but of similar mode size than Calu‐3 (Figure 1b). These EVs contained ACE2 protein as well as known EV markers (CD63, CD81 ADAM10) (Figure 1c). In addition, although Caco‐2 and Calu‐3 expressed TMPRSS2, this protease was not released in EVs (Figure 1c). To obtain EVs with high amounts of ACE2 and TMPRSS2 to be tested as decoy agents, we switched to 293FT cells that could be easily genetically‐edited. We transduced 293FT cells with lentivirus containing ACE2 alone (293FT‐ACE2) or in combination with TMPRSS2 (293FT‐ACE2‐TMPRSS2). 293FT cells transduced with lentivirus containing empty plasmids were used as a control (293FT‐mock). The three 293FT cell lines were cultured in FBS‐containing EV‐depleted medium and EVs were isolated from concentrated CCM by SEC. We observed a high count of particles with comparable mode sizes in EV fractions from all 293FT cell lines (Figure 1d, Supplementary Figure A) coincident with the presence of CD63, CD81, ADAM10 and HSP70 EV markers (Figure 1e). Overexpression of ACE2 in 293FT cells led to the incorporation of this molecule into EVs (Figure 1e) with higher levels found on EVs from 293FT‐ACE2 than from 293FT‐ACE2‐TMPRSS2 (Supplementary Figure B). 293FT‐mock and 293FT‐ACE2 cells expressed TMPRSS2, but the predicted full‐length 54 kDa form was not detected in their EVs: a 30 kDa N‐terminal fragment devoid of the serine protease domain (Zmora et al., 2015) was mainly detected, together with a low level of the glycosylated full‐length 70 kDa form. Importantly, EVs from 293FT cells overexpressing TMPRSS2 contained higher levels of the full‐length TMPRSS2 protein and less of a cleaved form than EVs from 293FT‐mock or 293FT‐ACE2 cells (Figure 1e, Supplementary Figure C) (Afar et al., 2001). Since the ACE2 ectodomain can be released after cleavage (Jia et al., 2009), we evaluated whether cells overexpressing ACE2 release soluble ACE2 in addition to EV‐associated ACE2. To do this, we analysed the presence of ACE2, the EV marker CD81 and the non‐EVs component AChE (Liao et al., 2019) by WB on EV fractions (1 × 10^9^   particles) and the intermediate and soluble fractions obtained from the same amount of CCM. We detected some particles in intermediate and soluble fractions from the three 293FT cell lines, that probably came from the depleted medium since they did not contain EV markers by WB (Supplementary Figure A, D, E). AChE was enriched in soluble fractions whereas CD81 was mainly found in EV fractions validating our isolation protocol for EVs and soluble components (Figure 1f). Importantly, ACE2 was found as a full‐length transmembrane form in the EV fractions, as two shorter cleaved forms in the soluble fractions, and a mixture of all forms in the intermediate fractions (Figure 1f). Intermediate fractions thus represent a mixture of EVs and soluble proteins and for this reason were not further analysed. Low levels of ACE2 were found in the soluble SEC fractions of 293FT‐ACE2 cells when compared to the levels present on the EV‐containing fraction (Figure 1f, 1g). By contrast, 293FT‐ACE2‐TMPRSS2 cells released similar amounts of EV‐associated as of soluble ACE2 (Figure 1f,1g), suggesting that the overexpression of the protease cleaves ACE2 as previously described (Heurich et al., 2014), favouring its secretion to the extracellular space as a soluble protein instead of associated to EVs.

**FIGURE 2:**
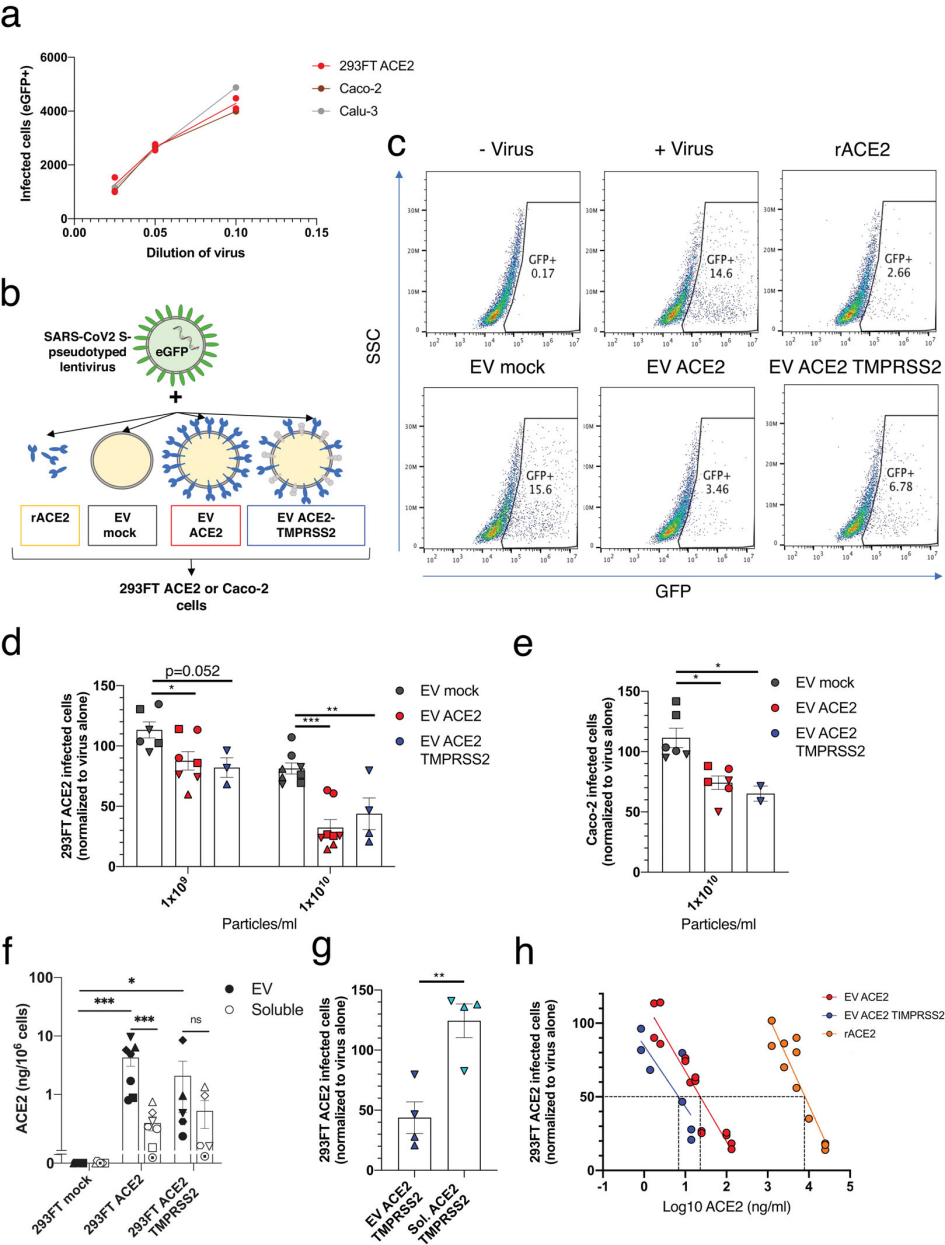
Inhibition of SARS‐CoV‐2‐S‐pseudotyped virus infection with ACE2 EVs. (a) Infection of 293FT‐ACE2, Caco‐2 and Calu‐3 cells with different dilutions of a SARS‐CoV‐2‐S‐pseudotyped lentivirus encoding for eGFP. The number of infected cells was calculated by multiplying the percentage of GFP‐positive cells by the initial number of cells. (b) Scheme of the infectivity assay with different treatments. (c) Dot plots showing the percentage of infected (= eGFP+) 293FT‐ACE2 cells obtained after incubation with viruses alone (0.05 dilution), in combination with 1 × 10^10^ 2020 EVs from the different 293FT cell lines or in combination with rACE2 (25 μg/ml). This rACE2 level represents 250 and 2960 times more ACE2 than the one contained in ACE2‐EVs and ACE2‐TMPRSS2‐EVs, respectively, measured by ELISA. (d) Quantification of the number of infected 293FT‐ACE2 cells in the presence of EVs. The percentage of eGFP+ cells was measured by FACS and normalized to infection with the virus alone (100%). Results from three independent experiments are shown. All replicates from each experiment are included. *P < 0.05; **P < 0.01; ***P < 0.001 (Dunnett's test). (e) Caco‐2 infection in the presence of ACE2‐EVs and ACE2‐TMPRSS2‐EVs. *P < 0.05 (Dunnett's test). (f) ACE2 quantification by ELISA in EV and soluble fractions obtained from the three different 293FT cell lines. Results are expressed as ng per million of secreting cells. *P < 0.05; ***P < 0.001 (non‐parametric ANOVA with Kruskal‐Wallis test for comparison among all groups). ***P < 0.001 (Mann‐Whitney test for comparison among EV vs soluble for each cell line). (g) Comparison of the effect on 293T‐ACE2 infection of 1 × 10^10^ 2020 EVs and soluble fractions from an equivalent volume of CCM of 293FT‐ACE2‐TMPRSS2 cells. **P < 0.01 (t‐test) (h) Percentage of infectivity from Figure 2d related to the amount of EV‐associated ACE2, quantified by ELISA in Figure 2f. As a comparison, cell infection rates in the presence of increasing amount of recombinant ACE2 (rACE2) were also determined (n = 4). Lines represent results of linear regression analysis. Comparison of slopes and intercepts using Prism indicated that the three regression lines are distinct but parallels. ACE2‐TMPRSS2 is different from ACE2 (*P* = 0.0017) and rACE2 is different from ACE2‐TMPRSS2 (*P* < 0.0001) and from ACE2 (*P* < 0.0001)

We then analysed the capacity of ACE2‐ and ACE2‐TMPRSS2‐containing EVs to reduce the infection of target cells by a lentivirus containing SARS‐CoV‐2‐S protein. The virus used in this study was produced using an HIV packaging vector pseudotyped with SARS‐CoV‐2 Spike protein and including a GFP‐coding sequence, which is expressed in infected cells. A variant of SARS‐CoV‐2 Spike protein bearing a region of the tail of the HIV gp41, instead of the Spike tail, previously shown to enhance infection of pseudotyped virus (Moore et al., 2004) was used. First, we determined the infectivity of the target cells Caco‐2, Calu‐3 and 293FT‐ACE2 by SARS‐CoV‐2‐S‐pseudotyped lentivirus and observed that each of these cell lines is infected similarly in a concentration‐dependent manner (Figure 2a). To assess the ability of ACE2‐containing EVs to decrease virus infectivity *in vitro*, we infected target cells with SARS‐CoV‐2‐S‐pseudotyped virus in the presence or the absence of EVs isolated from 293FT‐mock (mock‐EVs) or 293FT‐ACE2 (ACE2‐EVs) or 293FT‐ACE2‐TMPRSS2 cells (ACE2‐TMPRSS2‐EVs) (Figure 2b). Infection of 293FT‐ACE2 cells in the presence of ACE2‐EVs and ACE2‐TMPRSS2‐EVs was reduced while infection remained unaffected by mock‐EVs (Figure 2c and quantification in 2d). Importantly, this inhibition was dependent on the dose of EVs (Figure 2d). Caco‐2 infection was also reduced in the presence of ACE2‐EVs and ACE2‐TMPRSS2‐EVs (Figure 2e). We then quantified by enzyme‐linked immunosorbent assay (ELISA) the amount of ACE2 released by these cell lines. As previously documented by western blot (Figure 1f and 1g), we observed that 293FT‐ACE2 cells released high levels of ACE2 mainly associated with EVs while 293FT‐ACE2‐TMPRSS2 cells released lower ACE2 levels that were equally distributed between EV and soluble fractions (Figure 2f). Strikingly, ACE2 in the soluble fractions from these latter cells could not inhibit SARS‐CoV‐2‐S‐pseudotyped virus infection as compared to a comparable amount of ACE2 associated with EVs (Figure 2g). When re‐plotting the results obtained in Figure 2d as a function of the absolute amount of ACE2 measured for these same samples by ELISA (Figure 2f), we observed that EVs from cells overexpressing the full length TMPRSS2 together with ACE2 were more efficient at inhibiting SARS‐CoV‐2‐S‐pseudotyped viral infection than those from cells overexpressing ACE2 alone (Figure 2h). Moreover, to achieve similar levels of inhibition of lentiviral infection as those observed with ACE2‐ or ACE2‐TMPRSS2‐EVs, 500 to 1500 times more of the soluble recombinant human ACE2 had to be used (Figure 2h) in accordance with previous publications (Monteil et al., 2020). Altogether, these findings highlight the increased efficiency of EVs containing full‐length ACE2 to inhibit SARS‐CoV‐2‐S‐pseudotyped viral entry when compared to the soluble protein alone. The enhanced efficiency of EVs from cells overexpressing TMPRSS2 could be due to the presence of TMPRSS2 together with ACE2, leading to fusion of the virus with the EV thus impairing their capacity to infect cells, and/or to other modifications of the EV composition induced by overexpression of TMPRSS2 in the EV‐secreting cells.

## 3 DISCUSSION

Our data demonstrate that EVs containing ACE2, alone or in combination with TMPRSS2, block SARS‐CoV‐2 Spike‐dependent infection in a much more efficient manner than soluble ACE2. Thus, ACE2‐EVs represent a potential versatile therapeutic tool to block not only SARS‐CoV‐2 infection but also other coronavirus infections that use the ACE2 receptor for host cell entry, such as SARS‐CoV (Li et al., 2003) and NL63 (H et al., 2005). Further studies to determine the efficacy of the ACE2/TMPRSS2‐EVs in experimental models of SARS‐CoV‐2 virus need to be conducted to validate their therapeutic use for COVID‐19, but also the lack of side‐effects. The use of engineered EVs as therapeutic agents has been proposed several years ago and is currently being explored in humans (Wiklander et al., 2019), suggesting that well‐designed EV therapeutics against COVID‐19 may be feasible to prevent initial infection or further internal dissemination of the virus, and thus reducing the virus burden and disease severity. However, as recently highlighted by the International Societies for EV (ISEV) and for Cellular Therapies (ISCT) (Börger et al., 2020), despite the urgency induced by the current pandemic, EV‐based therapeutic developments for COVID‐19 will have to meet as strong criteria of manufacturing processes, quality controls and compliance to safety regulation as any other therapies, before they can be implemented in human subjects.

Note: a speculative article discussing the idea that we demonstrate experimentally here was published while we were preparing this article, thus showing concomitant emergence of similar scientific ideas (Inal, 2020).

## 4 METHODS

### 4.1 Cells

Human Caco‐2 (HTB‐37) and Calu‐3 (HTB‐55) were purchased from ATCC and maintained at 37°C in a humidified atmosphere with 5% CO_2_. Caco‐2 and Calu‐3 cells were cultured in DMEM (Thermo Fisher Scientific) supplemented with 10% FBS (Gibco), 100 U/ml penicillin‐streptomycin (Thermo Fisher Scientific) and 1% non‐essential amino acids (Thermo Fisher Scientific). For Calu‐3 cells the medium was also supplemented with 1 mM sodium pyruvate (Thermo Fisher Scientific) and 10 mM HEPES (Thermo Fisher Scientific). 293FT cells were cultured in DMEM medium (Thermo Fisher Scientific) supplemented with 10% FBS (Eurobio) and 100 U/ml penicillin‐streptomycin (Thermo Fisher Scientific). 293FT‐mock, 293FT‐ACE2 and 293FT‐ACE2‐TMPRSS2 cells were generated by stable double transduction with pTRIP‐SFFV‐tagBFP‐2A and pTRIP‐SFFV‐TagRFP657‐2A, pTRIP‐SFFV‐tagBFP‐2A‐hACE2 and pTRIP‐SFFV‐TagRFP657‐2A, or pTRIP‐SFFV‐tagBFP‐2A‐hACE2 and pTRIP‐SFFV‐TagRFP657‐2A‐TMPRSS2, respectively.

### 4.2 Plasmids

The plasmids psPAX2, CMV‐VSVG, pTRIP‐SFFV–tagBFP‐2A (Cerboni et al., 2017) and pTRIP‐SFFV‐eGFP‐NLS (Raab et al., 2016) were previously described. pTRIP‐SFFV‐TagRFP657‐2A was generated by PCR from a synthetic gene coding for TagRFP657. pTRIP‐SFFV‐tagBFP‐2A‐hACE2 and pTRIP‐SFFV‐TagRFP657‐2A‐TMPRSS2 constructs were obtained by PCR from pLenti6‐hACE2‐BSD (hACE2 sequence from Addgene #1786 subcloned into pLenti6‐BSD) and pCSDest‐TMPRSS2 (Addgene #53887) respectively. A codon optimized version of the SARS‐CoV‐2 S gene (GenBank: QHD43416.1) was transferred into the phCMV backbone (GenBank: AJ318514), by replacing the VSV‐G gene (phCMV‐SARS‐CoV‐2‐Spike) (Grzelak et al., 2020). phCMV‐SARS‐CoV‐2‐S‐H2 was obtained by PCR from phCMV‐SARS‐CoV‐2‐Spike in order to include the membrane‐proximal region of the cytoplasmic domain of HIV‐1 gp160 (NRVRQGYS, amino acid sequence) (Mammano et al., 1995) after residue 1246 of the S protein (Moore et al., 2004).

### 4.3 Preparation of EV‐depleted medium

EV‐depleted medium was obtained by overnight ultracentrifugation of DMEM supplemented with 20% FBS at 100,000 x *g* in a Type 45 Ti rotor (Beckman Coulter, K‐factor 1042.2). After ultracentrifugation, EV‐depleted supernatant was carefully pipetted from the top and leaving 7 ml in the bottom. Supernatant was filtered through a 0.22 μm bottle filter (Millipore) and additional DMEM and antibiotics were added to prepare complete medium (10% EV‐depleted FBS medium).

### 4.4 EV isolation by size‐exclusion chromatography

239FT‐mock, 293FT‐ACE2 and 293FT‐ACE2‐TMPRSS2 cells were cultured in FBS EV‐depleted medium for 24 h. Caco‐2 and Calu‐3 cells were cultured in FBS‐free DMEM for 24 h. Cell conditioned medium (CCM) was harvested by pelleting cells at 350xg for 5 min at 4°C three times. Supernatant was centrifuged at 2,000xg for 20 min at 4°C to discard 2K pellet and concentrated on a MiIlipore Filter (MWCO = 10 kDa, UCF701008) to obtain concentrated CCM. Medium was concentrated to 1 ml from 12 to 41 ml for Caco‐2 and Calu‐3 and from 75 ml for 293FT cells and overlaid on a 70 nm qEV size‐exclusion column (Izon, SP1). 0.5 ml fractions were collected and EVs were recovered in fractions 7 to 11 following manufacturer's instructions. We additionally collected intermediate fractions 12 to 16 and soluble factors in fractions 17 to 21. The three pools of fractions were concentrated using 10 kDa filter (Amicon, UCF801024) to reach a final volume of approximately 100 μl. Samples were stored aliquoted at ‐80°C.

We have submitted all relevant data of our experiments to the EV‐TRACK knowledgebase (EV‐TRACK ID: EV200117) (Van Deun et al., 2017).

### 4.5 Nanoparticle tracking analysis

NTA was performed to analyse EV fractions, intermediate fractions and soluble fractions using ZetaView PMX‐120 (Particle Metrix) with software version 8.04.02. The instrument was set a sensitivity 77 and shutter of 70. Measurements were done at 11 different positions (three cycles per position) and frame rate of 30 frames per second.

### 4.6 Western blotting (WB)

Cell lysate was prepared using lysis buffer (50 mM Tris, 150 mM NaCl, 1% Triton, pH 8) supplemented with Protease Inhibitor Cocktail (Sigma) at a concentration of 4 × 10^6^   cells in 100 μl of buffer. After incubation for 20 min on ice, samples were centrifuged at 18,500 × *g* for 20 min. The pellet was discarded and the supernatant was kept for further analysis. Cell lysates, EVs and the other SEC fractions were resuspended in Laemmli Sample Buffer (Bio‐Rad). Cells lysates corresponding to 4 × 10^5^   cells, the number of particles indicated in figures legends for EV fractions and the intermediate and soluble fractions obtained from the same volume of conditioned medium were loaded on 4%–15% Mini‐Protean TGX Stain‐Free gels (Bio‐Rad), under non‐reducing conditions. Transferred membranes (Immuno‐Blot PVDF Bio‐Rad) were incubated with antibodies and developed using Clarity Western ECL substrate (Bio‐Rad) and the ChemiDoc Touch imager (Bio‐Rad). Antibodies for WB were anti‐human: ACE2 (clone EPR4435 against extracellular domain, Abcam 108252), TMPRSS2 (clone EPR3681 against cytoplasmic domain, Abcam 92323), ADAM10 (clone 163003, R&D Systems MAB1427), CD63 (clone H5C6, BD Bioscience 556019), Syntenin‐1 (clone C2C3, Genetex GTX10847), CD81 (clone 5A6, Santa Cruz sc‐23692), HSP70 (clone C92F3A‐5, Enzo LifeScience, ADI‐SPA‐810‐D) and AChE (Abcam, ab31276). Secondary antibodies included HRP‐conjugated goat anti‐rabbit IgG (H+L) (Jackson 111‐035‐144), goat anti‐mouse IgG (H+L) (Jackson 111‐035‐146) and donkey anti‐goat IgG (Jackson, 705–035‐147).

### 4.7 Viral production

SARS‐CoV‐2‐S‐pseudotyped lentiviruses were produced by transient transfection of 293FT cells in 150 cm^2^ flask with 5 μg phCMV‐SARS‐CoV‐2‐S‐H2, 13 μg psPAX2 and 20 μg pTRIP‐SFFV‐eGFP‐NLS and 114 ul of TransIT‐293 (Mirus Bio). SARS‐CoV‐2‐S‐pseudotyped viruses’ supernatant was centrifuged at 300xg for 5 min to remove dead cells, filtered with a 0.45 μm filter (Millipore) and loaded on top of a 20% sucrose gradient for concentration. Viral concentration was achieved by ultracentrifugation at 120,000 x *g* for 1 h 30 min at 4 °C in a SW32i rotor. The pellet containing concentrated SARS‐CoV‐2 S‐pseudotyped virus from three 150 cm^2^ flasks was resuspended in 1 ml EV‐depleted DMEM and 100 μl aliquots were stored at ‐80°C.

### 4.8 Infectivity assay

20,000 Caco‐2 or Calu‐3 cells or 10,000 293FT‐ACE2 cells were seeded in a 96 well plate 6 h before infection with SARS‐CoV‐2 S‐pseudotyped virus. Infection was performed in the absence or in the presence of different amount of EVs or human recombinant ACE2 (Abcam, 151852) by spinoculation at 1200 x *g* for 1 h 30 min at 25°C. 48 h after infection, cells were washed, trypsinized and fixed. Infection was measured by analysing eGFP expression using a CytoFLEX LX cytometer. Data were analysed using FlowJo software.

### 4.9 ACE2 enzyme‐linked immunosorbent assay

Quantification of the amount of human ACE2 in the different EV preparations and other SEC fractions was done using the human ACE2 ELISA kit (Abcam, ab235649) following manufacturer's instructions.

## AUTHOR CONTRIBUTIONS

Federico Cocozza, Ester Piovesana, Nathalie Névo, Xavier Lahaye performed the experiments. Federico Cocozza, Ester Piovesana, Mercedes Tkach, Lorena Martin‐Jaular, Clotilde Théry analysed the data. Lorena Martin‐Jaular, Clotilde Théry, Mercedes Tkach designed the experiments. Ester Piovesana, Federico Cocozza, Mercedes Tkach, Clotilde Théry, Lorena Martin‐Jaular wrote the paper. Xavier Lahaye, Nicolas Manel, Julian Buchrieser, Olivier Schwartz designed plasmids. Xavier Lahaye, Nicolas Manel generated cells overexpressing ACE2 and TMPRSS2 and developed the infection assay.

## Supporting information


**Supplementary Figure. Isolation and characterization of EVs containing ACE2 and TMPRSS2** (A) NTA quantification of the particles produced by 10^6^ cells contained in pools of SEC fractions for one (Caco‐2/Calu‐3) or several (293FT) independent isolations. Error bars indicate SEM. (B, C) Quantification of signals for ACE2 and TMPRSS2 in WB from Figure 1E. (B) ACE2 signal (pooled full‐length and cleaved forms) in 3–4 independent WB. AU = arbitrary units after background subtraction (C) TMPRSS2 signal ration of full length (pooled 54 KDa + glycosylation forms) over cleaved (2 pooled forms) in 3 independent experiments. (D) Number of particles counted in each fraction of 293FT‐mock, 293FT‐ACE2 and 293FT‐ACE2‐TMPRRS2 CCM, compared with isolation of non‐conditioned depleted medium performed in parallel. Numbers of particles are normalized to the volume of medium used for each purification. (E) WB analysis of non‐conditioned depleted medium fractions isolated by SEC (material loaded on the WB was obtained from 15 ml initial volume of depleted medium for EV, 1.5 ml for intermediate and 0.75 ml for soluble).
